# Activation of Locus Coeruleus‐Hippocampus Tyrosine Hydroxylase Projection Contributes to the Surgical Incision Pain‐Induced Memory Consolidation Enhancement in Mice

**DOI:** 10.1111/cns.70570

**Published:** 2025-09-04

**Authors:** Xin Qing, Jinyang Liu, Yinyao Feng, Luyao Zhang, Jiawei Sun, Peng Wang, Zhilai Yang, Jiqian Zhang, Hu Liu, Xuesheng Liu

**Affiliations:** ^1^ Department of Anaesthesiology, The First Affiliated Hospital of Anhui Medical University, Key Laboratory of Anaesthesia and Perioperative Medicine of Anhui Higher Education Institutes Anhui Medical University Hefei China; ^2^ Department of Medicine Southern University of Science and Technology Shenzhen China

**Keywords:** hippocampus, locus coeruleus, memory consolidation, postoperative post‐traumatic stress disorder, surgical incision pain

## Abstract

**Aims:**

The mechanism underlying postoperative post‐traumatic stress disorder (PTSD) remains unclear. However, studies have shown that acute postoperative pain is an independent risk factor for PTSD, which is also closely related to memory consolidation enhancement. Preoperative patients often experience unpleasant traumatic events, and postoperative pain usually occurs in the memory consolidation stage of these events. Therefore, inquiring whether acute postoperative pain affects memory consolidation and its possible mechanism may help to explain the causes of postoperative PTSD.

**Methods and Results:**

In this study, we show that the surgical incision pain enhances the consolidation of emotional memory (in the passive avoidance test) and nonemotional memory (in the novel object recognition test) in mice. None of the behaviors evaluated were affected by anxiety or locomotor dysfunction (in the open‐field test). Besides, we confirmed that surgical incision pain promotes memory enhancement by enhancing memory consolidation instead of memory retrieval. Furthermore, the consolidation of emotional memory and nonemotional memory was enhanced by the activation of the LC‐HPC TH projection after surgical incision pain. Hippocampal CA1 dopamine receptors, rather than β adrenoceptors, mediate emotional and nonemotional memory consolidation enhancement after surgical incision pain.

**Conclusion:**

Thus, our results indicate that surgical incision pain enhances the memory consolidation of emotional memory and nonemotional memory in mice. Activation of the LC‐HPC TH projection may contribute to memory consolidation enhancement induced by surgical incision pain, which involves the activity of dopamine receptors in CA1.

## Graphical abstract

Surgical incision pain enhances the consolidation of both emotional and nonemotional memory in mice, likely through activation of the LC‐HPC TH projection, which releases dopamine and acts on the D1/5 receptors in the CA1 region.

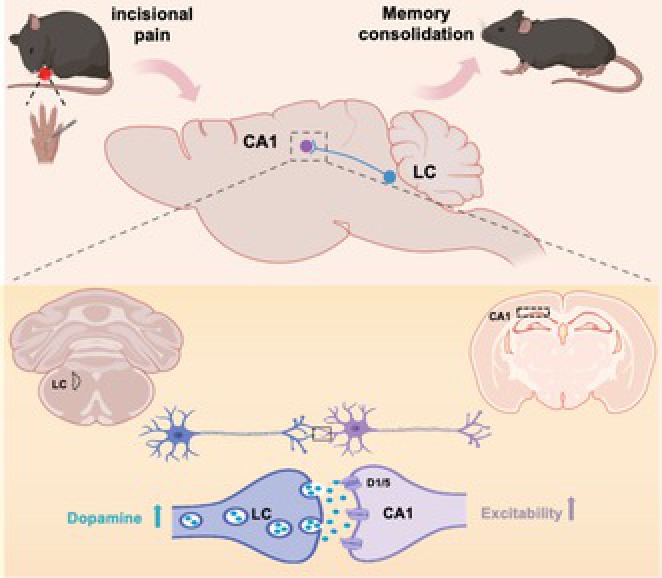

## 1 Introduction

Clinical evidence shows that approximately 20% of patients develop post‐traumatic stress disorder (PTSD) after surgery [1]. This rate is significantly higher than the estimated lifetime rate of 6.8% and the estimated annual prevalence of 3.5% for PTSD within the general population [2, 3]. Furthermore, PTSD seriously impacts physical health and mental comorbidity [4], increasing the risk of suicide and economic burden [5]. Given that over 320 million surgeries are performed annually worldwide and the exact mechanism of postoperative PTSD is still unclear [6]. Therefore, it is urgent to understand the causes and mechanisms of postoperative PTSD.

PTSD is characterized by the obvious enhancement of fear memory, which may result from the strengthening of memory encoding and consolidation or the weakening of memory extinction [7]. Previous studies have shown that anesthetic drugs, such as propofol, could enhance emotional memory consolidation immediately after stressful events, potentially contributing to the occurrence of postoperative PTSD [8, 9]. However, the risk factors for postoperative PTSD include not only anesthetic drugs but also the surgery itself, preoperative psychological disorders, intraoperative awareness, and postoperative pain [10]. In fact, patients after emergency surgery often experienced acute postoperative pain. Nearly 20% of patients experienced severe pain in the first 24 h after the operation, and this figure has remained basically unchanged in the past 30 years [11]. Therefore, acute postoperative pain may be a major cause of postoperative PTSD. Moreover, studies have shown that acute postoperative pain is an independent risk factor for PTSD, closely related to enhancing memory consolidation [10, 12]. Therefore, it is worth exploring whether acute postoperative pain promotes memory consolidation, thereby leading to postoperative PTSD.

According to current theories, pain and cognition interact reciprocally, sharing common neural matrixes [13]. Notably, fear memory engram cells in the prefrontal cortex have been shown to perpetuate chronic pain [14]. Furthermore, peripheral nerve injury impairs locus coeruleus (LC) noradrenergic modulation of CA1 astrocytic lactate release, disrupting pyramidal neuron excitability and contributing to comorbid hyperalgesia and memory deficits [15]. The LC is involved in regulating various physiological functions, including arousal [16], depression [17], memory consolidation [18] and pain [19]. It serves as the primary source of norepinephrine (NE) and dopamine (DA) in the central nervous system, with extensive projections to the amygdala [19], prefrontal cortex (PFC) [20] and hippocampus (HPC) [21]. Previous studies have demonstrated that chronic pain enhances emotional memory in mice by enhancing norepinephrine projection from LC‐amygdala [22]. Converging ex vivo and in vivo evidence further demonstrates that TH^+^ neurons in the LC enhance memory retention through corelease of dopamine and noradrenaline in the hippocampus [18, 21, 23]. Mechanistically, activation of LC‐HPC TH projection enhances memory consolidation by increasing synaptic related proteins [24]. Importantly, acute pain and stress are known to activate neurons in LC [25, 26], suggesting that the LC‐HPC TH projection may serve as a potential neural mechanism for pain‐related memory enhancement.

Based on these findings, we hypothesize that acute stress conditions, such as surgical incisional pain, could activate LC neurons, leading to increased release of norepinephrine and/or dopamine in the hippocampus, thereby potentiating hippocampus‐dependent memory consolidation. This could provide a possible explanation for the high incidence of PTSD observed postoperatively, linking acute postoperative pain to the neural mechanisms underlying memory consolidation.

## 2 Methods

### 2.1 Animals

The animal care and use in this study strictly adhered to institutional guidelines and government regulations. All experiments were conducted in accordance with the approved regulations of Anhui Medical University. Efforts were made to minimize the number of animals used in this study.

The following strains of mice were used: Th‐IRES‐Cre: (B6;129‐Th^tm1(cre/Esr1)Nat^/J) were donated by Professor Wang Liping from Shenzhen Institute of Advanced Technology and C57BL/6J mice (8–14 weeks, male) purchased from GemPharmatech Co. Ltd. Mice were housed under a 12‐h light/dark cycle (light from 8:00 a.m. to 8:00 p.m.), with a maximum of 5 mice per cage, allowing for socialization. Mice have free access to water and food. Environmental conditions were maintained at a temperature of 22°C–25°C and a relative humidity of 50%–60%. All experiments were strictly randomized and double‐blind. The animal protocol was approved by the Laboratory Animal Ethics Committee of Anhui Medical University, Hefei, Anhui. All experimental procedures were performed in accordance with the National Institutes of Health Guide for the Care and Use of Laboratory Animals (NIH Publication No. 8023, revised 1978).

### 2.2 Surgical Incision Pain Model

In this study, the surgical incision pain model was implemented according to the previous literature [27, 28, 29]. Immediate pain induction aligns with the peak memory consolidation window, while 6‐h induction tests retrieval‐specific effects [30, 31, 32, 33]. The mice were anesthetized with 2% sevoflurane for 3 min and then transferred to the experimental operating table with an insulating blanket. The body temperature of the mice was maintained at 37°C and 2% sevoflurane was continuously inhaled. After sterilizing the right paw of the mouse with iodophor, the skin and fascia of the right plantar foot of the mice were incised longitudinally using a No. 10 sterile scalpel blade, and the length of the incision was approximately 0.5 cm. The incision begins 0.2 cm from the proximal edge of the heel and extends distally. Use curved forceps to elevate the muscles of the sole and make longitudinal incisions (3 times). The skin covering the muscle is then sutured with 5.0 nylon mattress sutures (the operation takes about 2 min). After surgery, the mice were returned to cages with sterile padding for recovery. Mice in the control group were anesthetized with 2% sevoflurane for 5 min. All surgeries were performed by the same experimenter to ensure consistency.

### 2.3 Behavioral Procedures

On the test day, mice were transferred to the testing room and allowed to acclimate to the room conditions for at least 1 h. After each individual test, carefully wipe the instrument with 70% alcohol to remove the residual odor or traces of the last mouse. To avoid potential interactions between behavioral tests, two separate cohorts of mice were used for the NORT and PAT.

#### 2.3.1 Novel Object Recognition Test (NORT)

The NORT was performed as described previously [34]. The novel object recognition training and testing in this experiment were conducted in the open‐field chamber. The diameter of the open field is 50 × 50 cm and the height is 40 cm. The experiment was carried out in 3 days. The first day was the adaptation stage, and the mice were placed in an open field to move freely for 10 min. The next day, a T‐25 flask filled with red ink (familiar object A) and a 50 mL centrifuge tube filled with water (familiar object B) were placed in the center of the open field. During novel object recognition training, the mice were allowed to explore two objects freely in the open field for 10 min, and the activities and exploration behavior of the mice were observed using ANY‐maze software (Stoelting Co., Wood Dale, IL, USA). After 24 h of training, the mice were returned to the open field again, and the familiar object B was replaced with a 10 cm high vase (novel object). Two experienced researchers watched the ANY‐maze software video and recorded the times of exploring old and novel objects during the 5‐min test period. The frequencies (*f*) of investigating objects A and C within 5 min are defined as the mouse's nose approaching the object by less than 2 cm. The object discrimination index (DI) was calculated as: (*f*_novel_ − *f*_familiar_)/(*f*_novel_ + *f*_familiar_) × 100%.

#### 2.3.2 Passive Avoidance Test (PAT)

The PAT was performed as described previously [35], with some modifications. The passive avoidance shuttle box consists of two parts: a black chamber and a white chamber. There is an autolifting central control door in the middle of the box. The bottom of the black and white chamber is composed of a conductive grid floor. The current size and electric shock time of the black and white chamber floor can be set separately. In the passive avoidance training, the mice were placed in the white chamber with their backs to the elevator door for 30 s of free exploration, and then, the elevator door was lifted. Since mice have a dark‐loving nature, they will quickly pass through the central control door and explore the black chamber. When the limbs of the mice completely entered the black box, 0.12–0.14 mA current stimulation was given until the mouse returned to the white box. After the mouse returned to the white box, the central control door was closed and the training was terminated. In this experiment, the trained mice that did not enter the black box within 40 s after the central control door was opened or did not return to the white box within 5 s after the electric shock were excluded. The purpose is to eliminate the situation that individual mice are insensitive to black boxes or have strong memories of the environment due to long‐term electrical stimulation. 24 h after training, the mice were placed in the white box with their backs facing the central control door, and the central control door was open at this time. Because the dark environment has been associated with electric shock during training, the latency of mice entering the black box from the white box reflects the memory strength of the mice. There was no electric shock stimulation in the black chamber, and the software recorded the latency of the mice entering the black chamber from the white chamber. After the mice entered the black chamber, the central control door closed and the experiment ended. The time of the memory retention test was 600 s. The latency period of mice that did not enter the black box within 600 s was recorded as 600 s.

#### 2.3.3 Open‐Field Test (OFT)

Mice underwent an open‐field test to assess their general motor abilities and anxiety‐like behaviors. Under dim lighting conditions, each mouse was gently placed into an open‐field box measuring 50 × 50 × 40 cm. During the test, each mouse was gently placed in the central area of the open field and allowed to move freely for 5 min. The camera recorded the activities of the mice in the open field. ANY‐maze software calculates the total distance traveled and the time spent in the central area of the open field.

#### 2.3.4 Von Frey Test

In this study, mechanical pain thresholds were assessed using a 2450 series electronic von Frey aesthesiometer (IITC 2091). Baseline measurements were obtained prior to surgical incision pain, followed by repeated assessments at 2, 4, and 6 h postincision to evaluate pain sensitivity changes. Before the test, mice were placed in plexiglass (5 × 5 × 8 cm) with a wire mesh floor to adapt for at least 1 h. Keep the environment quiet and the temperature suitable (18°C–22°C) during adaptation. The bottom of the plexiglass plate is a mesh plate made of iron wire. During the measurement, the soft needle tip of the electronic von Frey tactile sensor was pricked at the sole of the hind paw of the mouse, and it was slowly lifted until the foot retraction reflex was observed. Record the value (Withdrawal threshold) displayed on the von Frey tactile instrument when the mouse has a foot contraction reflex. Each mouse was measured three times with an interval of 5 min each time, and the average of the three paw withdrawal thresholds was calculated.

### 2.4 Viral Vectors

All the adeno‐associated viruses (AAVs), including rAAV‐EF1α‐DIO‐mCherry, rAAV‐EF1α‐DIO‐hM3D(Gq)‐mCherry, rAAV‐EF1α‐DIO‐hM4D(Gi)‐mCherry, and retro‐AAV2‐hsyn‐DIO‐mCherry, were obtained from Shanghai Shengbo Biomedical Technology Co. Ltd. AAVs were stored at −80°C until use. The concentration of virus used for stereotaxic injection was 5 × 10^12^ vg/mL.

### 2.5 Stereotaxic Viral Injections

Mice were anesthetized with pentobarbital sodium (100 mg/kg, i.p.) and fixed in a stereotactic frame (RWD Life Science, Shenzhen, China). The skin was cut from the middle of the scalp to expose the skull surface, and then, holes were drilled at the corresponding positions on the flat skull surface. A pulled glass microelectrode was backfilled with virus and connected to a microsyringe pump (RWD Life Science, Shenzhen, China); the injection volume of different viruses varied from 200 to 300 nL depending on the viral titer and expression potential, and the infusion rate was 40 nL/min. The locations of the locus coeruleus and hippocampal CA1 area were determined according to references [36, 37]. The site was defined using the following coordinates: Locus coeruleus: anterior posterior (AP) from bregma: −5.45 mm, medial lateral (ML) from the midline: ±1.25 mm, dorsal ventral (DV) from the brain surface: −3.65 mm; hippocampus CA1: AP from bregma: −2.18 mm, ML from the midline: ±1.18 mm, DV from the brain surface: −1.36 mm. According to different experiments, different viruses were injected into the locus coeruleus. Virus varieties include the anterograde tracer virus rAAV‐EF1α‐DIO‐mCherry, as well as rAAV‐EF1α‐DIO‐hM3D(Gq)‐mCherry and rAAV‐EF1α‐DIO‐hM4D(Gi)‐mCherry, which were used in chemogenetic experiments. The viruses and drugs injected into the hippocampus include retrograde non‐transsynaptic tracer viruses retro‐AAV2‐hsyn‐DIO‐mCherry and CNO (Enzo Life Sciences, USA). To ensure the virus spread evenly, the glass electrode was drawn 10 min after the virus injection. After the injection, the mouse scalp was sutured. The injection protocol of virus and CNO was based on previous research methods [38]. In chemogenetic experiments and hippocampal adrenoceptor/dopamine receptor blockade experiments, to block the TH^+^ projection activity of the locus coeruleus‐hippocampus, CNO (1 μg/μL/side), adrenergic receptor [18] (propranolol, China, Macklin, 3.125 μg/0.5 μL/side), and dopamine receptor blockers [39] (SCH23390, UK, Abcam, 0.5 μg/0.5 μL/side) were administered through a microdosage system. Behavioral tests were conducted 1 week after recovery from cannula implantation.

### 2.6 Immunofluorescence

Mice were deeply anesthetized with pentobarbital sodium (100 mg/kg, i.p.) and followed sequentially by transcardiac perfusion with ice‐cold phosphate‐buffered saline (PBS, 0.1 M, pH 7.4) and 4% paraformaldehyde (PFA). The brains were removed and postfixed in 4% PFA at 4°C overnight and then incubated in 30% sucrose solution overnight for dehydration. Brains were embedded in optimal cutting temperature (OCT) compound (Sakura, Japan) and immediately stored at −80°C, and then, 40 μm coronal sections containing the LC and CA1 were cut using a freezing microtome (MNT, SLEE, Mainz, Germany). Brain slices were permeabilized with 0.3% Triton X‐100 (BS084, Biosharp, Hefei, China) in PBS for 30 min and blocked with 5% bovine serum albumin (BSA) in PBS for 1 h. The brain slices were then incubated with the primary antibodies at 4°C overnight. Primary antibodies used were anti‐TH (1:1000, mice, Abcam), anti‐c‐Fos (1:1000, rabbit, Synaptic system). After washing, followed by corresponding fluorophore‐conjugated secondary antibodies including donkey anti‐rabbit Alexa Fluor 647 (1:1000, Jackson ImmunoResearch), donkey anti‐mouse Alexa Fluor 488 (1:1000, Jackson ImmunoResearch) at 37°C for 1 h in the dark and exposed to DAPI (BL105A, Biosharp, Hefei, China) in the dark at room temperature for 10 min. After washing, the sections were sealed on microscope slides with anti‐fluorescence quenching sealed tablets (BL701A, Biosharp, Hefei, China) and stored at 4°C before analysis. Fluorescence signals were visualized using a Zeiss LSM800 confocal microscope.

### 2.7 In Vitro Electrophysiological Recordings of Brain Slice

In vitro electrophysiological experiments, we injected the retrograde tracer virus retro‐AAV2‐hsyn‐DIO‐mCherry into the CA1 region of 8–12‐week‐old TH‐Cre mice. 4–5 weeks after the virus has fully infected the neurons, the mice were anesthetized with sodium pentobarbital (100 mg/kg, intraperitoneal injection), and then perfused transcardially with ice‐cold oxygenated (95% O_2_, 5% CO_2_) N‐methyl‐Dglucamine artificial cerebrospinal fluid (ACSF). The perfusate formulation includes a solution of 93 mM N‐methyl‐Dglucamine (NMDG), 2.5 mM potassium chloride (KCl), 1.25 mM monosodium phosphate (NaH_2_PO_4_), 10 mM magnesium sulfate (MgSO_4_), 93 mM hydrogen chloride (HCl), 25 mM glucose, 30 mM sodium bicarbonate (NaHCO_3_), 20 mM HEPES, 3 mM sodium pyruvate (C_3_H_3_NaO_3_), 5 mM sodium ascorbate (C_6_H_7_O_6_Na) and 2 mM thiourea (CH_4_N_2_S). After sufficient perfusion, the mouse brain was quickly decapitated and transferred to ice‐cold NMDG ACSF solution. The rat brain in buffer was sliced into coronal sections at a thickness of 300 μm using a vibrating microtome (VT1200 S, Leica, Germany). The mouse brain slices containing the locus coeruleus area were transferred to oxygenated NMDG ACSF, incubated at 32°C for 15 min, and then transferred to ACSF (2.5 mM KCl, 1.25 mM NaH_2_PO_4_, 126 mM NaCl, 10 mM glucose, 2 mM MgSO_4_, 2 mM CaCl_2_ and 26 mM NaHCO_3_) for 1 h at room temperature. All chemicals used in section preparation were purchased from Sigma‐Aldrich, USA.

The prepared mice brain slices were transferred to the electrophysiological recording room, and then ACSF was slowly perfused into the recording room at a rate of 3 mL/min at 28°C. TH^+^ neurons in the Locus coeruleus were identified microscopically using a differential interference optical microscopy imaging system (Olympus BX61WI, Japan). Recording electrodes (3–4 MΩ) were prepared using a micropipette puller (P2000, Sutter Instrument, USA). For whole‐cell recording, the recording electrode was filled with ACSF solution (18 mM NaCl, 133 mM C_6_H_11_KO_7_, 0.6 mM EGTA, 2 mM Mg·ATP, 10 mM HEPES and 0.3 mM Na3·GTP (pH: 7.2, 280 mOsm)). After establishing whole‐cell recording, hold the neuron at 0 pA in current clamp mode to record spontaneous action potentials from TH^+^ neurons. Before drug infusion, a baseline of at least 3 min of spontaneous action potentials was recorded. Electrophysiological data were acquired using a Multiclamp 700B amplifier, with the signal low‐pass filtered at 3 kHz and digitized at 10 kHz using the DigiData 1550 system from Molecular Devices, USA. Data analysis was conducted using Clampfit 10 software (Molecular Devices, USA) and the Mini Analysis Program (Synaptosoft, USA).

### 2.8 Statistical Analysis

All sample sizes were selected according to previous research using similar experimental paradigms, and all statistical analyses were carried out by using GraphPad Prism 7.0. For measurement data, the normality test is conducted first. D'Agostino and Pearson tests or Shapiro–Wilk tests were used to examine the normal distribution of the data. Data conforming to a normal distribution are represented by mean ± SD, while non‐normally distributed data are expressed as median and interquartile range (25%–75%).

For comparisons between independent experimental groups in postoperative NORT, OFT, patch‐clamp recording data, and immunofluorescence quantification, an unpaired two‐tailed Student's *t*‐test was employed. A paired two‐tailed Student's *t*‐test was applied to analyze patch‐clamp recordings obtained before and after pharmacological interventions. The nonparametric Kruskal‐Wallis test followed by Bonferroni‐adjusted post hoc analysis was utilized for pairwise comparisons in the PAT. In chemogenetic manipulation and pharmacological validation experiments, intergroup differences in NORT were assessed using one‐way ANOVA with post hoc Bonferroni's multiple‐comparison correction. For mechanical pain thresholds, two‐way repeated measures ANOVA with Bonferroni post hoc testing was implemented. Statistical significance was defined as *p* < 0.05 for all analyses.

## 3 Results

### 3.1 Surgical Incisional Pain Enhances Emotional and Nonemotional Memory Consolidation in Mice

**FIGURE 1:**
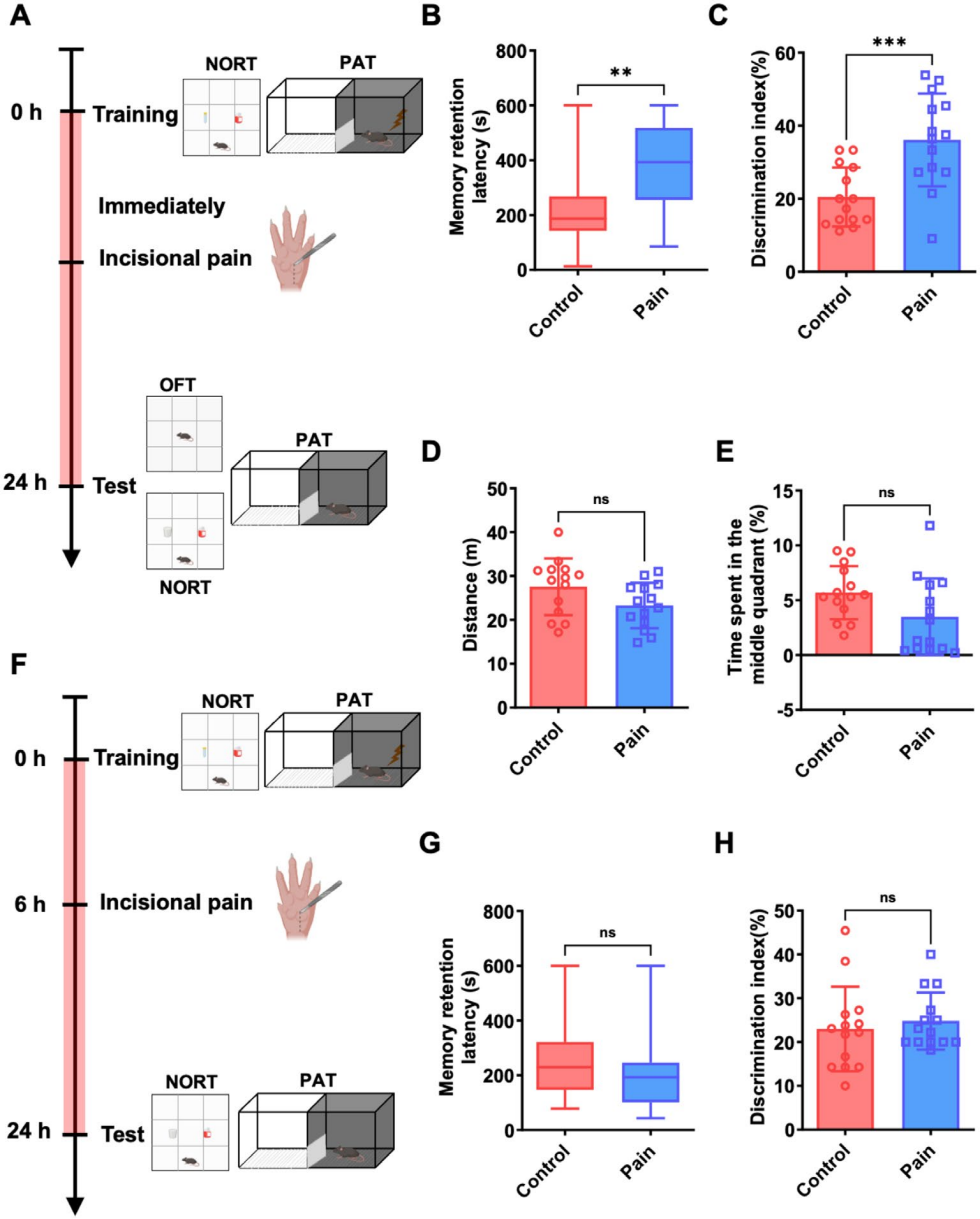
Surgical incision pain enhances emotional and nonemotional memory consolidation in mice. (A) Schematic diagram of surgical incision pain performed immediately after behavioral training to estimate the effect of surgical incision pain on memory consolidation. (B) Surgical incision pain performed immediately after training enhanced the escape latency in the PAT. (C) Surgical incision pain performed immediately after training enhanced the object discrimination index in the NORT. (D) Distance traveled in the OFT after 24 h of surgical incision pain. (E) Percentage of time spent in the center area in OFT after 24 h of surgical incision pain. (F) Schematic diagram of surgical incision pain performed 6 h after behavioral training to estimate the effect of surgical incision pain on memory retrieval. (G) Surgical incision pain performed 6 h after training did not affect the escape latency in the PAT. (H) Surgical incision pain performed 6 h after training did not affect the object discrimination index in the NORT. Data are presented as the mean ± SD or median values (and interquartile ranges), **p < 0.01, ***p < 0.001, *n* = 14 per group. HPC, hippocampus; NORT, novel object recognition test; OFT, open‐field test; PAT, passive avoidance test.

In the present study, we investigated the effect of surgical incision pain on the particular stages of memory consolidation. Novel object recognition test (NORT) and passive avoidance test (PAT) were conducted to evaluate nonemotional memory and emotional memory, respectively. The surgical incision pain model was established immediately after behavioral training, and memory retention tests were conducted 24 h after training (Figure 1A). The step‐through latency (latency to enter the dark compartment) of pain mice was significantly longer than that of the control mice in the PAT (Figure 1B; 187.0 s (142.8–267.5 s), median and interquartile range (25%–75%), vs. 392.5 s (255.5–517.8 s), *p* < 0.05, *n* = 14 mice). Similarly, the pain mice exhibited a higher discrimination index in the NORT compared to the control mice (Figure 1C; 20.5% ± 8.1% vs. 36.1% ± 12.7%, *p* < 0.05, *n* = 14 mice). Besides, the open‐field test (OFT) results at 24 h postsurgery demonstrated no statistically significant difference in the total distances traveled and the percentage of time spent in the middle quadrant between the control and pain mice (Figure 1D,E; total distances: 27.6 ± 6.5 m vs. 23.3 ± 5.2 m, time spent in the middle quadran: 5.7% ± 2.4% vs. 3.5% ± 3.5%, *p* > 0.05, *n* = 14 mice), suggesting that the pain mice showed no anxiety or locomotor dysfunction. These results indicated that surgical incisional pain enhances emotional and nonemotional memory consolidation in mice, independent of anxiety‐related behaviors or locomotor impairment.

The memory retention test conducted 24 h after behavioral training inherently involves memory retrieval. Given that surgical incision pain persists for at least 7 days postmodeling [28, 40], it is plausible that surgical incision pain could also enhance memory by interfering with memory retrieval during the retention test. To specifically investigate whether surgical incision pain enhances memory by affecting memory retrieval, we performed a surgical incision pain 6 h after behavioral training to assess memory retention, thereby minimizing the potential influence of surgical incision pain on memory consolidation (Figure 1F). As is depicted in Figure 1G, the control mice and pain mice exhibited comparable performance in the PAT [Figure 1G; 229.5 s (146.8–321.5 s), median and interquartile range (25%–75%), vs. 192.5 s (101.3–245.8 s), *p* > 0.05, *n* = 14 mice]. In the NORT, the novel object discrimination index did not significantly differ between control mice and the pain mice (Figure 1H; 23.0% ± 9.7% vs. 24.8% ± 6.5%, *p* > 0.05, *n* = 14 mice). These results suggest that memory enhancement induced by surgical incision pain is not by affecting memory retrieval in mice but through enhancing memory consolidation.

### 3.2 Determination of the Anatomical Connection Between LC and HPC

**FIGURE 2:**
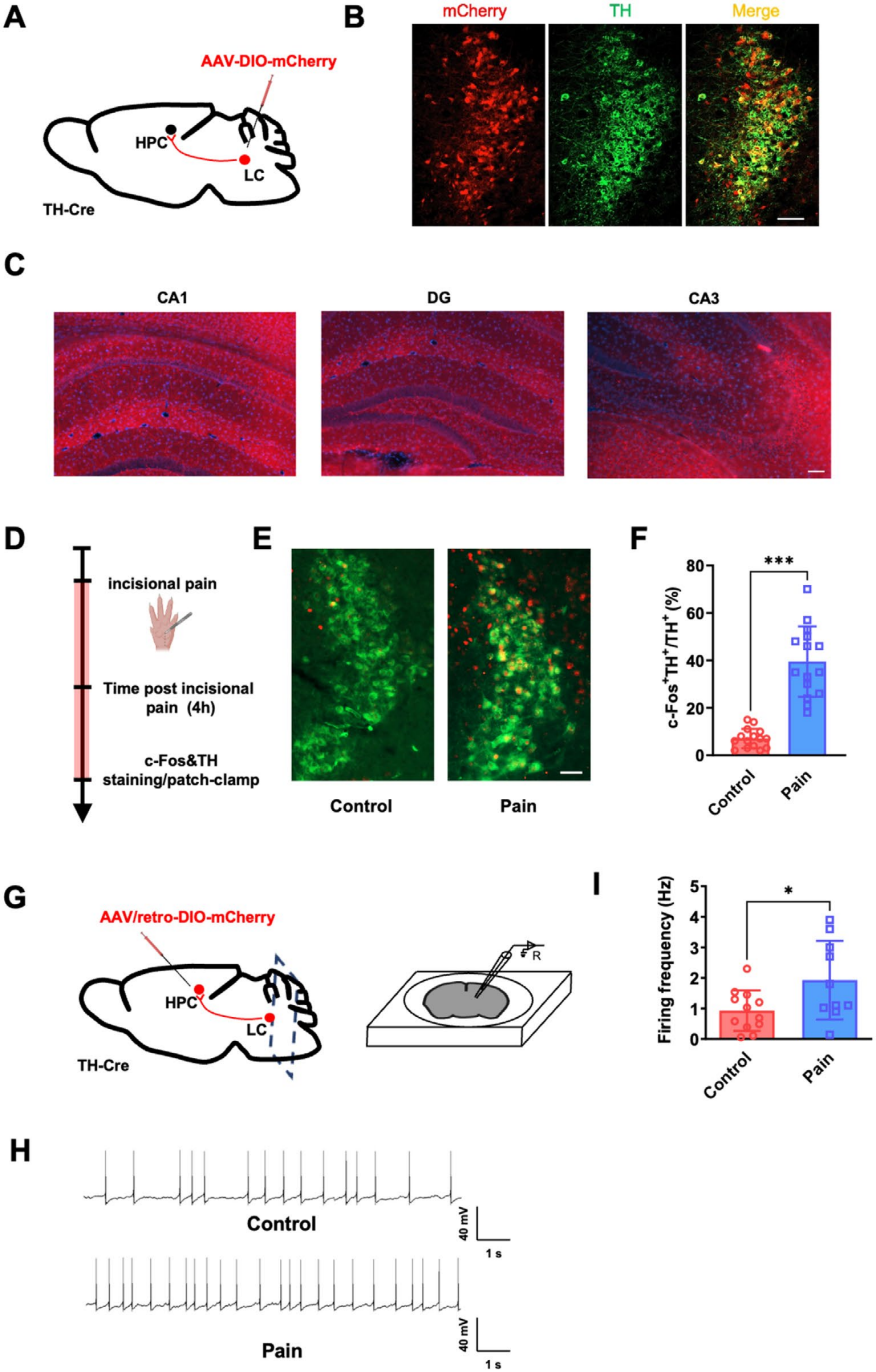
Surgical incision pain enhances the activity of LC‐HPC TH^+^ neurons. (A) Schematic of viral injection. (B) Representative immunohistochemistry of mCherry expression in LC neurons, Scale bars: 100 μm (red = mCherry, green = tyrosine hydroxylase). (C) Representative image (CA1, DG and CA3 from one slice) shows robust anterograde labeling of the HPC from injection in the LC, Scale bars: 200 μm. (D) Scheduling of c‐Fos staining 4 h after surgical incision pain. (E) Representative immunohistochemistry of c‐Fos positive neurons in control and surgical incision pain animals, Scale bars: 100 μm. (F) Quantification of c‐Fos positive neurons in the LC, *n* = 15 slices from 4 mice. (G) Schematic of virus tracing strategy and patch‐clamp recording. (H) Sample traces showing spontaneous firing events of TH neurons in LC, Scale bars: 40 mV, 1 s. (I) Statistical analysis of the spontaneous firing frequency of LC‐HPC TH^+^ neurons, *n* = 10–12 neurons from 4 mice. Data are presented as the mean ± SD, *p < 0.05, ***p < 0.001. HPC, hippocampus; LC, locus coeruleus; TH, tyrosine hydroxylase.

Our findings demonstrate that surgical incision pain can enhance emotional memory and nonemotional memory consolidation in mice. To investigate the role of LC—HPC TH projection in memory consolidation enhancement after surgical incision pain, we injected rAAV‐EF1α‐DIO‐mCherry based on Cre recombinase expression into LC in TH‐Cre strain mice (Figure 2A). After virus expression, the fluorescent mCherry protein was successfully expressed in LC neurons and co‐labeled with TH^+^ neurons (Figure 2B). TH neuron fiber terminals from LC were detected in CA1, DG, and CA3 regions of the hippocampus (Figure 2C). The above results confirm the TH projection from the LC to the HPC.

We next examined the effect of surgical incision pain on the LC—HPC TH projection (Figure 2D). Our results revealed that the number of TH^+^ neurons labeled for c‐Fos in LC increased significantly 4 h after surgical incision pain (Figure 2E,F; 7.1% ± 4.1% vs. 39.5% ± 14.9%, *p* < 0.05, *n* = 15 slices from 4 mice). To determine that the TH neurons activated in LC are specifically projected to HPC, we injected retrograde tracer virus (retro‐AAV2‐hsyn‐DIO‐mCherry) into HPC in TH‐Cre strain mice and recorded the excitability changes of mCherry‐labeled TH neurons in LC using whole‐cell patch clamp (Figure 2G). As shown in Figure 2H,I, the firing rate of TH neurons showed a significant increase after surgical incision pain (0.9 ± 0.7 Hz vs. 1.9 ± 1.3 Hz, *p* < 0.05, *n* = 10–12 neurons from 4 mice). These results demonstrate that surgical incision pain enhances the activity of LC TH neurons projecting to HPC.

### 3.3 Chemogenetic Inhibition of LC‐HPC TH Projection Prevents Surgical Incision Pain‐Induced Memory Consolidation Enhancement

**FIGURE 3:**
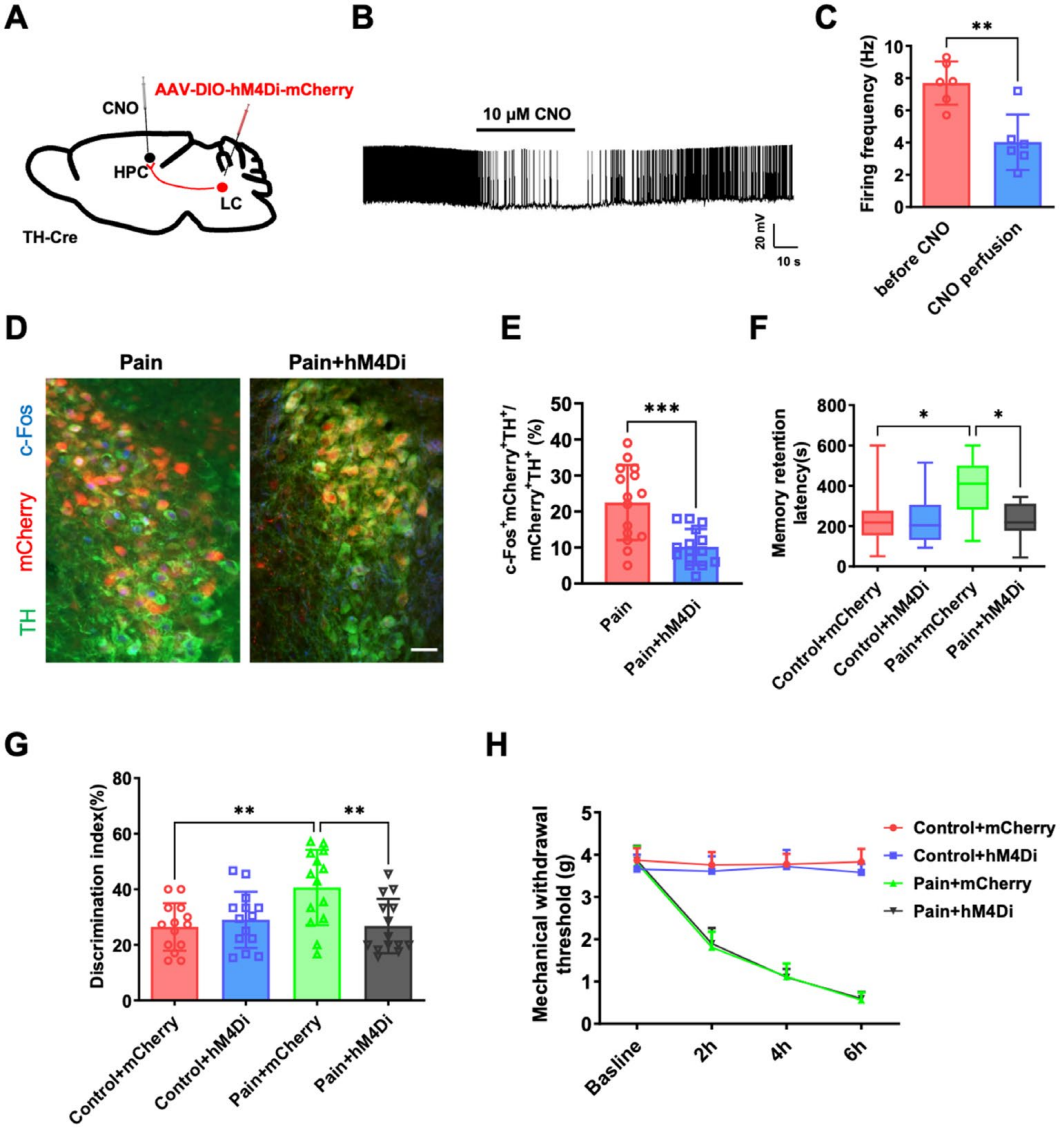
Chemogenetic inhibition of LC‐HPC TH^+^ projection prevents surgical incision pain‐induced memory consolidation enhancement. (A) Schematic of viral injection. (B) Sample traces of spontaneous firing events induced by CNO recorded from LC mcherry^+^ TH neurons in acute brain slices, Scale bars: 20 mV, 10 s. (C) Statistical analysis of the spontaneous firing frequency induced by CNO recorded from LC mcherry^+^ TH neurons in acute brain slices, *n* = 6 neurons from 6 mice. (D) Representative confocal images showing that c‐Fos and TH labeled neurons in the LC of surgical incision pain mice predominantly colocalized with the mCherry, Scale bars: 100 μm. (E) Quantification of c‐Fos positive neurons in the LC, *n* = 15 slices from 4 mice. (F) The effects of inhibiting the LC‐HPC TH^+^ projection on memory performance in the PAT, *n* = 14 mice. (G) The effects of inhibiting the LC‐HPC TH^+^ projection on memory performance in the NORT, *n* = 14 mice. (H) The mechanical pain tests were carried before and 2, 4, 6 h after surgical incision pain to evaluate the effects of inhibiting the LC‐HPC TH^+^ projection, *n* = 14 mice. Data are presented as the mean ± SD or median values (and interquartile ranges), *p < 0.05, **p < 0.01, ***p < 0.001. CNO, clozapine‐N‐oxide; HPC, hippocampus; LC, locus coeruleus; NORT, novel object recognition test; PAT, passive avoidance test; TH, tyrosine hydroxylase.

To investigate whether the activity of the LC‐HPC TH projection is involved in the memory consolidation enhancement after surgical incision pain, we used a clozapine‐N‐oxide (CNO)‐based chemogenetic inhibition approach by injecting the Cre‐dependent rAAV‐EF1α‐DIO‐hM4Di‐mCherry into the LC of TH‐Cre mice (Figure 3A). The whole‐cell patch clamp recording confirmed that CNO administration significantly reduced the firing rate of LC TH neurons, indicating successful inhibition of TH neurons activated by pain (Figure 3B,C; 7.7 ± 1.3 Hz vs. 4.0 ± 1.7 Hz, *p* < 0.05, *n* = 6 neurons from 6 mice). Four weeks post‐Avirus injection, we established the surgical incision pain model and administered CNO (1 mg/kg i.p.) immediately postsurgery. CNO treatment reduced the ratio of mCherry and c‐Fos co‐labeled positive neurons in pain mice (Figure 3D,E; 22.5% ± 10.4% vs. 10.1% ± 5.0%, *p* < 0.05, *n* = 15 slices from 4 mice). In behavioral experiments, Cre‐dependent rAAV‐EF1α‐DIO‐hM4Di‐mCherry virus or rAAV‐EF1α‐DIO‐mCherry virus was injected into the LC. Immediately after the surgical incision pain model was established, we administered CNO (1 μg/μL/side) into the hippocampal CA1 area through the cannula. As shown in Figure 3F, the step‐through latency of pain mice (Pain + mCherry) increased significantly in comparison to the control mice (Con + mcherry) in the PAT [Figure 3F; 218.5 s (154.5–275.5 s), (25%–75%), vs. 410.0 s (282.5–500.5 s), *p* < 0.05, *n* = 14 mice]. However, compared with the pain mice (pain + mCherry), the step‐through latency of hM4Di mice (pain + hM4Di) was significantly reduced in the PAT [Figure 3F; 410.0 s (282.5–500.5 s), median and interquartile range (25%–75%), vs. 218.0 s (177.5–311.0 s), *p* < 0.05, *n* = 14 mice]. In the NORT, the discrimination index of pain mice (Pain + mCherry) was significantly higher than that of control mice (Con + mcherry; Figure 3G; 26.4% ± 8.5% vs. 40.6% ± 13.5%, *p* < 0.05, *n* = 14 mice), and the discrimination index of hM4Di mice (pain + hM4Di) was lower than the pain mice (Pain + mCherry; Figure 3G; 40.6% ± 13.5% vs. 26.8% ± 9.8%, *p* < 0.05, *n* = 14 mice). These results indicate that surgical incision pain leads to memory consolidation enhancement by activating the LC‐HPC TH projection, while inhibiting the LC‐HPC TH projection reversed the memory consolidation enhancement induced by surgical incision pain. Studies have suggested that noradrenergic neuronal activation in the LC mediates initiating and maintaining pain [17, 41]. Therefore, after the LC‐HPC pathway was inhibited by CNO for 6 h, the pain hypersensitivity was evaluated by Von Frey. The results showed that inhibition of the LC‐HPC TH projection had no effect on pain in 2–6 h (Figure 3H, *p* > 0.05，*n* = 14). These findings indicate that the alleviation of surgical incision pain‐induced memory consolidation enhancement through inhibition of LC TH neurons projecting to the HPC is not due to pain relief, but due to a direct modulation of memory‐related neural circuits.

### 3.4 Chemogenetic Activation of LC‐HPC TH Projection Enhanced Memory Consolidation

**FIGURE 4:**
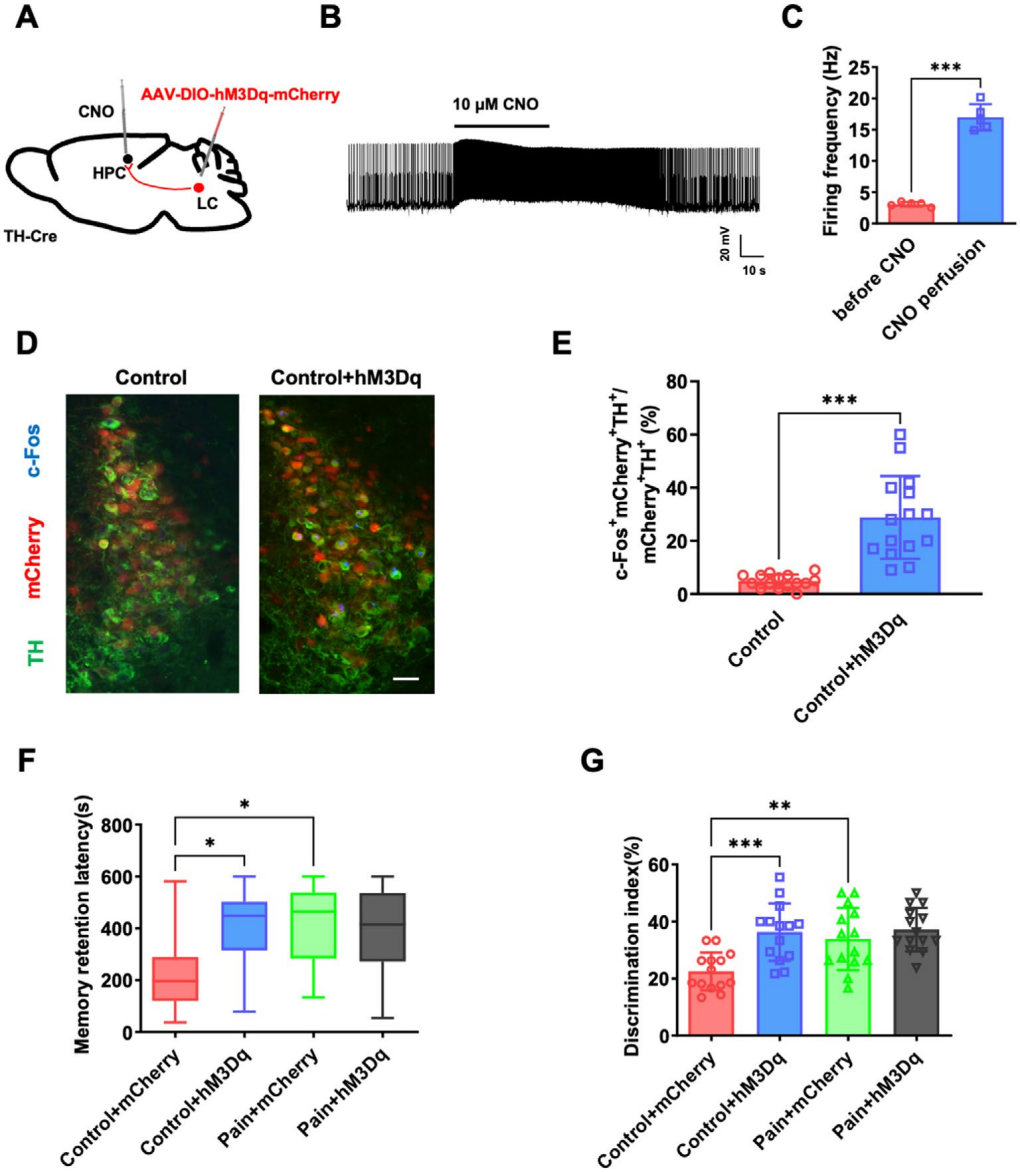
Chemogenetic activation of LC‐HPC TH^+^ projection enhanced memory consolidation. (A) Schematic of viral injection. (B) Sample traces of spontaneous firing events induced by CNO recorded from LC mcherry^+^ TH neurons in acute brain slices, Scale bars: 20 mV, 10 s. (C) Statistical analysis of the spontaneous firing frequency induced by CNO recorded from LC mcherry^+^ TH neurons in acute brain slices, *n* = 5 neurons from 5 mice. (D) Representative confocal images showing that c‐Fos and TH labeled neurons in the LC of surgical incision pain mice predominantly colocalized with the mCherry, Scale bars: 100 μm. (E) Quantification of c‐Fos positive neurons in the LC, *n* = 15 slices from 5 mice. (F) The effects of activation the LC‐HPC TH^+^ projection on memory performance in the PAT, *n* = 14 mice. (G) The effects of activation the LC‐HPC TH^+^ projection on memory performance in the NORT, *n* = 14 mice. Data are presented as the mean ± SD or median values (and interquartile ranges), *p < 0.05, **p < 0.01, ***p < 0.001. CNO, clozapine‐N‐oxide; HPC, hippocampus; LC, locus coeruleus; NORT, novel object recognition test; PAT, passive avoidance test; TH, tyrosine hydroxylase.

To further clarify the role of LC‐HPC TH projection in memory consolidation, the mice were stereotaxically injected in the LC with either the hM3Dq virus or a control virus (Figure 4A). After hM3Dq expression, whole‐cell patch clamp recordings revealed that CNO administration significantly increased the firing rate of LC TH neurons, suggesting chemical inheritance effectively activated the TH neurons in LC (Figure 4B,C; 3.1 ± 0.4 Hz vs. 17.0 ± 2.1 Hz, *p* < 0.05, *n* = 5 neurons from 5 mice). In addition, immunofluorescence results further confirmed that the expression of mcherry and c‐Fos co‐labeled neurons increased significantly by CNO administration (Figure 4D,E; 4.8% ± 2.5% vs. 28.8% ± 15.6%, *p* < 0.05, *n* = 15 slices from 4 mice). As shown in Figure 4F, hM3Dq mice (Con + hM3Dq) and pain mice (pain + mcherry) exhibited a longer step‐through latency than the control mice (Con + mcherry) in the PAT [Figure 4F; 196.5 s (120.8–288.5 s), median and interquartile range (25%–75%), vs. 448.5 s (315.5–502.0 s) and 465.0 s (284.3–537.8 s), *p* < 0.05, *n* = 14 mice]. In the NORT, the discrimination index of pain mice (pain + mcherry) was higher than control mice (con + mcherry; Figure 4G; 22.5% ± 6.7% vs. 33.9% ± 10.9%, *p* < 0.05, *n* = 14 mice). hM3Dq mice (Con + hM3Dq) also exhibited a higher discrimination index relative to the control mice (Con + mcherry; Figure 4G; 22.5% ± 6.7% vs. 36.3% ± 10.1%, *p* < 0.05, *n* = 14 mice). These results indicate that activation of LC‐HPC TH projection can enhance emotional memory and nonemotional memory consolidation in mice, effectively recapitulating the memory‐enhancing effects observed following surgical incision pain.

### 3.5 HPC D1/5 Receptors Are Involved in Surgical Incision Pain‐Induced Memory Consolidation

**FIGURE 5:**
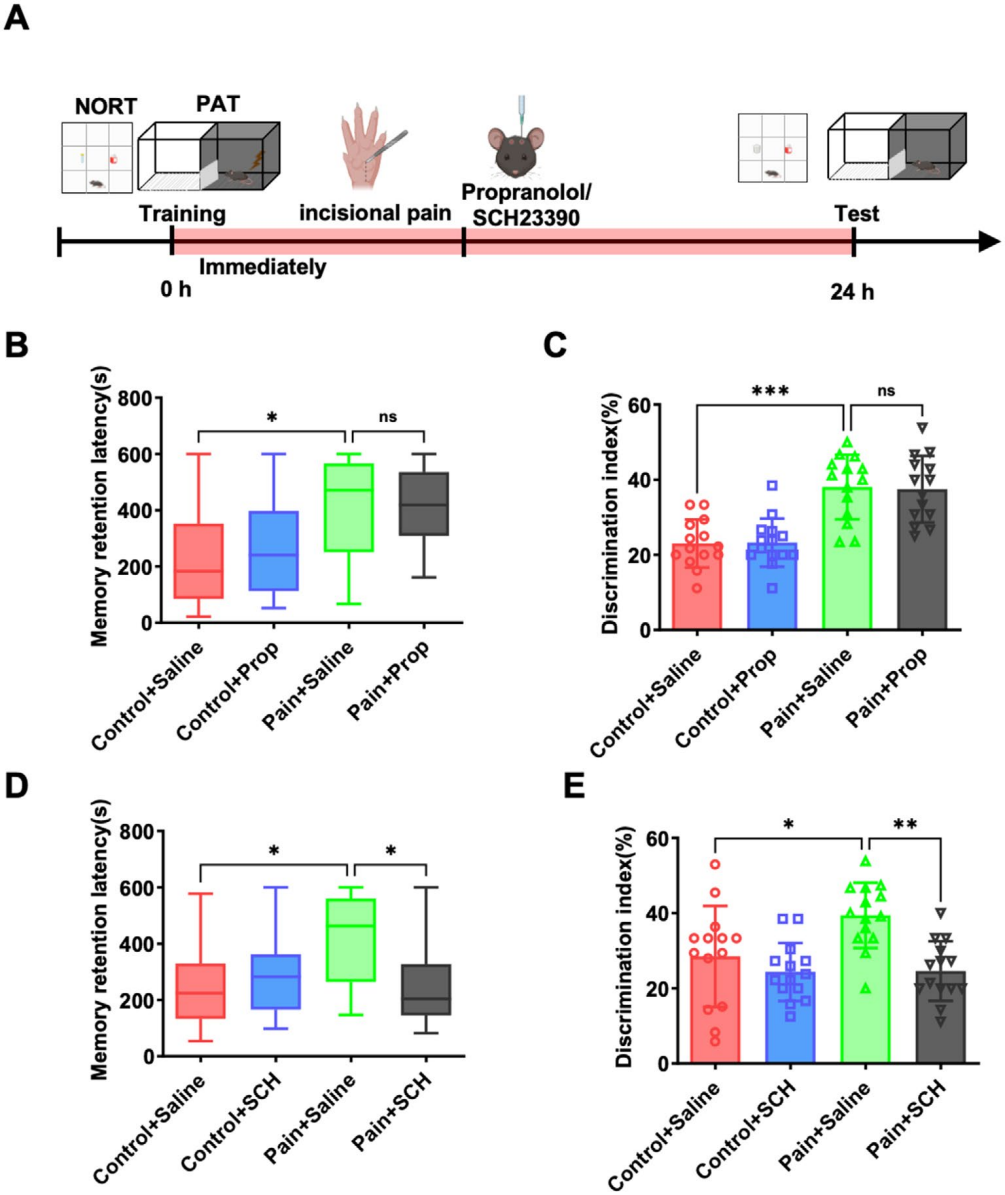
HPC D1/5 receptors are involved in surgical incision pain‐induced memory consolidation. (A) Schematic of the effect of surgical incision pain on memory consolidation. (B) The effects of inhibition of the β‐ARs in the hippocampus on memory performance in the PAT. (C) The effects of inhibition of the β‐ARs in the hippocampus on memory performance in the NORT. (D) The effects of inhibition of the D1/5 receptors in the hippocampus on memory performance in the PAT. (E) The effects of inhibition of the D1/5 receptors in hippocampus on memory performance in the NORT. Data are presented as the mean ± SD or median values (and interquartile ranges), *p < 0.05, **p < 0.01, ***p < 0.001, *n* = 14 per group. NORT, novel object recognition test; PAT, passive avoidance test.

LC‐HPC TH projection activation releases two neurotransmitters, norepinephrine and dopamine [21]. To explore which neurotransmitter is involved in memory consolidation enhancement induced by surgical incision pain, we microinjected β adrenoceptor antagonist (propranolol) and dopamine receptor antagonist (SCH23390) into the CA1 (Figure 5A). The selection of β‐adrenergic receptor antagonists rather than α‐adrenergic antagonists was due to their established role in hippocampal memory consolidation [42, 43]. As shown in Figure 5B, the step‐through latency of the pain group (Pain+Sal) and propranolol group (Pain+Prop) did not significantly differ in the PAT. [Figure 5B; 471.0 s (250.8–566.0 s), median and interquartile range (25%–75%), vs. 418.5 s (309.0–535.8 s), *p* > 0.05, *n* = 14 mice]. Similarly, in the NORT, there was no significant difference in the discrimination index between the pain group (Pain + Sal) and propranolol group (Pain + Prop; Figure 5C; 38.1% ± 8.6% vs. 37.5% ± 8.9%, *p* > 0.05, *n* = 14 mice). These results indicated that propranolol could not reverse the memory consolidation enhancement caused by surgical incision pain. On the contrary, in the PAT, SCH23390 significantly reversed the increased step‐through latency induced by surgical incision pain [Figure 5D; 462.5 s (264.3–559.8 s), median and interquartile range (25%–75%), vs. 204.0 s (145.5–327.3 s), *p* < 0.05, *n* = 14 mice]. The increased discrimination index of the pain group (Pain+Sal) in the NORT was significantly reduced by SCH23390 administration (Figure 5E; 39.4% ± 8.7% vs. 24.6% ± 7.9%, *p* < 0.05, *n* = 14 mice). These data indicate that blocking the D1/5 receptor in the hippocampus reverses the memory consolidation enhancement induced by surgical incision pain.

## 4 Discussion

Previous studies showed that acute postoperative pain is an independent risk factor for PTSD, which is closely related to memory consolidation enhancement [10, 12]. Patients often experience traumatic events or unpleasant treatment experiences before surgery, and the pain caused by surgery often occurs in the memory consolidation stage of those events. Therefore, clarifying the effect of acute postoperative pain on memory consolidation may help to explain the reason for PTSD caused by acute postoperative pain. In this study, we aim to study the effect of surgical incision pain on memory consolidation by behavioral experiments, virus tracing, chemogenetics, and electrophysiological recording. Our findings suggest that surgical incision pain enhanced emotional and nonemotional memory consolidation in mice and the activity of LC‐HPC TH projection. Chemogenetic inhibition of LC‐HPC TH projection effectively attenuates memory consolidation enhancement of emotional and nonemotional memories induced by surgical incision pain. Our results also suggest that memory consolidation enhancement induced by surgical incisional pain may be mediated by activation of the LC‐HPC TH projection, which releases dopamine and acts on the D1/5 receptor in the CA1 region. In addition, our results show that surgical incision pain does not cause locomotor dysfunction and anxiety, which is consistent with the previous research results [27]. The memory enhancement after surgical incision pain is not through enhancing memory retrieval but by enhancing memory consolidation. Our research results also showed that the mechanical pain threshold of mice is significantly decreased 2–6 h after surgical incision pain, consistent with the previous study [40]. However, selective chemogenetic inhibition of LC‐HPC TH projection did not relieve surgical incision pain. These results indicate that LC‐HPC TH projection was not involved in the regulation of pain, and chemogenetic inhibition of LC‐HPC TH projection reduced the memory consolidation enhancement, not by relieving surgical incision pain.

The LC and HPC are important brain regions involved in learning and memory [44, 45]. In this study, our most important finding is that the memory consolidation enhancement after surgical incision pain is mediated by activating the LC‐HPC TH projection. Previous studies have shown that the LC‐HPC TH projection releases norepinephrine and dopamine at the same time [45, 46]. However, our results show that surgical incision pain causes memory enhancement by activating the LC‐HPC TH projection, which releases dopamine to HPC and acts on dopamine receptors (D1/5 receptor) in CA1. In fact, norepinephrine is also an important transmitter for memory consolidation [47, 48]. Although our results only proved that dopamine contributes to the memory consolidation enhancement after surgical incision pain. Indeed, norepinephrine is one of the main transmitters involved in the memory consolidation enhancement [47, 48]. Previous studies have shown that activation of norepinephrine receptors in the DG region enhances memory consolidation in rats after passive avoidance training [49]. However, our results showed that only dopamine was involved in the enhancement of memory consolidation after surgical incision pain, which may be due to the uneven density distribution of noradrenergic axons in various subregions of the rat hippocampus. For example, the density of noradrenergic axons in the DG region was higher than that in the CA1 region [18, 21, 50]. Therefore, the memory consolidation enhancement may act through norepinephrine receptors in the DG region and dopamine receptors in the CA1 region. However, we cannot rule out that surgical incision pain mediates memory consolidation by activating β‐adrenergic receptors in DG or CA3. Confirming this speculation needs further study.

At present, there are a few studies about the influence of acute pain on learning and memory. In this study, we found pain enhanced memory consolidation, which is consistent with previous studies. For example, Li et al. showed that activating LC‐projecting ACC neurons facilitates pain‐evoked aversive consolidation and memory, providing novel insights into the treatment of pain‐related emotional aversions [26]. However, there are some conflicts with other previous studies. For example, Xie et al. found that surgical incision pain increased the TNFα and CDK5 levels in the cortex, thus reducing the expression of the NMDA2B receptor in the synapse, which further damaged the spatial and situational memory in 9‐month‐old mice [27]. We suspect that the reason why our results are different from those of Xie et al. is probably that Xie et al. conducted behavioral training immediately after surgical incision pain, which may interfere with memory coding, thus damaging spatial and emotional memory. In addition, Xie et al. used 9‐month‐old mice in the experiment, while we used 8–14‐week‐old mice. The effect of surgical incision pain on memory in mice may be age‐dependent. Ian N. Johnston et al. [51] observed that subcutaneous injection of formalin 1 h after learning and training can reduce consolidation of hippocampus‐independent conditioned fear memory in rats. Besides, Mayla K. Lazzarim's [52] research also found that formalin injection into the sole can damage the memory consolidation of rats, and the pain caused by formalin has no effect on memory encoding and memory retrieval. However, the pain models and animal species used in our study are different from the above two studies. In addition, in order to completely cover the whole process of memory consolidation, our research plan chooses the time point of surgical incision pain modeling as immediately after learning and training, not 1 h after training. Moreover, the pain caused by formalin usually lasts for several days, and Ian N. Johnston's research did not rule out the possible influence of formalin on memory retrieval. Although Mayla K. Lazzarim's research showed that formalin did not affect the memory retrieval of rats, the pain model, animal species, and behavioral methods used in their research were different from this study, which leads to the different results of the two studies. Nevertheless, we have revealed that pain enhances memory consolidation, which may explain the occurrence of postoperative PTSD.

In this study, we first revealed the neural circuit mechanism of surgical incision pain enhancing memory consolidation, but there are still some limitations. First of all, previous studies have shown that other LC brain targets (hippocampus, the amygdala, and the cortex) involved in learning and memory [53]. Our study does not exclude the possibility that surgical incision pain through LC and other brain region projections caused memory consolidation enhancement. Secondly, we only used β‐adrenergic and dopamine receptor antagonists to prove the effect of dopamine and norepinephrine on memory consolidation after surgical incision pain. The high‐performance liquid chromatography (HPLC) or neurotransmitter probes could help to verify the change of dopamine and norepinephrine in CA1 after incision pain. Additionally, in the context of pain‐related memories, impaired extinction could lead to the persistence of maladaptive memories, similar to those observed in PTSD [54]. Future investigations should explore whether surgical incision pain impairs memory extinction and whether this contributes to the long‐term persistence of pain‐related memories. Finally, we used c‐Fos staining and in vitro whole‐cell patch clamp to verify the effect of surgical incision pain on the activity of LC‐HPC TH projection. Although c‐Fos can be used as an indicator of neuronal activation [55, 56, 57], as an early gene, it is usually expressed 1–4 h after neuronal excitation, which has a time‐delay effect. Therefore, c‐Fos cannot dynamically reflect the real state of surgical incision pain on TH neurons in LC. The optical fiber‐based calcium signal recording and two‐photon calcium imaging in vivo can more accurately detect the transient changes of LC TH neurons after surgical incision pain.

Although there are some shortcomings in this study, the results first prove that the activation of LC‐HPC TH projection mediates the consolidation of emotional memory and nonemotional memory induced by surgical incision pain, which involves the activity of dopamine receptors in CA1. Our results indicate that acute pain in the perioperative period may lead to PTSD after operation by enhancing memory consolidation.

## 5 Conclusion

In summary, this study demonstrated that surgical incision pain enhances the consolidation of emotional memory and nonemotional memory in mice. Memory consolidation enhancement induced by surgical incision pain may be due to activation of the LC‐HPC TH projection, which releases dopamine and acts on the D1/5 receptor in the CA1 region. This study provides new insights into the mechanism of postoperative PTSD and offers a new target for future treatment of PTSD.

## Author Contributions

Experiment design: H.L., X.L.; patch‐clamp electrophysiology: X.Q.; Behavioral tests: X.Q., H.L.; histology and immunostaining: X.Q., Y.F.; data collection/analysis: J.L., J.S., P.W., J.Z., Z.Y.; writing of the paper: H.L., J.L., L.Z. All authors have read and approved the final manuscript.

## Conflicts of Interest

The authors declare no conflicts of interest.

## Data Availability

The data that support the findings of this study are available from the corresponding author upon reasonable request.
